# Effects of health insurance on non-working married women’s medical care use and bed days at home

**DOI:** 10.1186/1472-6963-13-243

**Published:** 2013-07-01

**Authors:** Changwoo Lee, Euichul Shin

**Affiliations:** 1 Korea Insurance Research Institute, 35-4 Yoido, Youngdeungpo, Seoul 150-606, Korea; 2 Department of Preventive Medicine, College of Medicine, Catholic University of Korea, Seoul 137-701, Korea

**Keywords:** Bed days at home, Medical care, Health insurance, Service use

## Abstract

**Background:**

This study examines whether bed days are alternative methods to medical care use for treating a particular illness. If bed days at home are considered as an alternative to medical treatment, then medical care use and bed days at home should be influenced by an individual’s health insurance status.

**Method:**

This study uses data from the 2003 Medical Expenditure Panel Survey (MEPS) on medical care use and bed days at home for each contracted illness of non-working married women.

**Results:**

The results suggest that the health insurance status of non-working married women has considerable influence on their choice between medical care use and bed days at home. In addition, those with health insurance are more likely to use medical care and less likely to use bed days at home, but they tend to avoid the simultaneous use of medical care and bed days at home.

**Conclusions:**

In contrast to previous studies’ findings indicating that absences from work and medical care use among working males may be complements, this study’s results for non-working married women without health insurance suggest that they use rest and medical treatment as substitutes, not complements.

## Background

US flu.gov webpage recommend getting a plenty of rest as a treatment on flu without medication [1]. It might be a small example but it show that people use taking a rest as one of treatments on their illness when they are sick. In this respect, if bed days at home are considered as an alternative to medical treatment, then medical care use and bed days at home should be influenced by an individual’s health insurance status. In particular, medical care use and bed days may be complements for individuals with health insurance because the latter can be interpreted as a type of alternative health care and cost less than medical care use. In addition, medical care use and bed days at home can be substitutes for individual without health insurance, who may prefer bed rest to hospital visits because bed days at home are less costly than medical treatment.

This study explores the effects of health insurance on the choice between medical care use and bed days at home to evaluate whether these are alternative methods for treating an illness. For this, the study employs the health insurance status as a proxy for the cost of medical care, which can influence medical care use and bed days. If health insurance plays a role in the decision on the medical use by affecting the relative price of medical care use, then a lack of insurance can lead to a preference for bed days at home over medical care. However, if health insurance does not influence the decision on bed days, then the decision on bed days at home may not be associated with the relative price of medical care use.

This study focuses on non-working married women because bed days at home for these individuals may represent “sick leave” days for those outside the labor market as a result of some illness. In addition, the health insurance status of non-working married women is more likely to be dependent on their spouse’s economic status than that of single or working women. Therefore, their health insurance status may be given, not selected, and is expected to play an important role in non-working women’s decision on medical care use and bed days at home as a proxy for the relative cost. Further, the effects of the cost of medical care use and ‘absences from work’ for non-working females may provide important implications for the relationship between medical care and “taking a rest” in terms of medical treatment.

This study employs the 2003 Medical Expenditure Panel Survey (MEPS) because this survey provides data on bed days at home and medical care use for each medical condition [2,3]. The dependent variables capture the following four choices: no medical care use or bed days; medical care use but no bed days; no medical care use but some bed days; and both medical care use and bed days. The study employs a longitudinal dataset because multiple observations over various medical conditions are nested within an individual. However, the number of medical conditions varies across individuals (i.e., an unbalanced panel). The panel dimension of the data is not over time but over the medical condition of each individual.

The study employs the maximum simulated likelihood (MSL) estimation to control for unobserved heterogeneity across individuals. If individuals become ill at Xrandom points in time, then each time an individual becomes ill, he or she has an opportunity to make another choice regarding medical care use and bed days at home. Therefore, this study assumes that the decision on medical care use and bed days at home for each illness is made repeatedly by the individual.

The rest of this paper is organized as follows: Section 'Method' provides a literature review. Section 'Data and descriptive statistics' presents the data set and descriptive statistics. Section 'Empirical framework' discusses this study’s theoretical background and empirical approach. Section 'Results and discussion' presents this study’s results and limitations, and Section 'Conclusions' concludes.

## Method

### Previous research

A number of studies have examined the relationship between medical care use and health insurance by focusing on the role of health insurance in medical care use. For this, these studies have generally controlled for the selection bias associated with the choice of health insurance because of the interaction between medical care use and health insurance decisions. The Rand Health Insurance Experiment (HIE) avoided the selection problem by randomly assigning each participating family to one of 14 health insurance plans [4]. Previous studies based on the HIE have estimated the sensitivity of healthcare demand to prices, income, and deductible and found that this demand depends on health insurance and that there exists moral hazard. On the other hand, traditional instrument variables represent the most popular method for solving the selection problem, but Goldman, Cardon and Hendel and Mello et al. set a joint distribution including commonly unobserved factors in health insurance decisions and healthcare demand [5-8]. As in Cardon and Hendel, Deb and Trivedi controlled for this selection bias by using latent factors influencing individuals’ medical care use and health insurance choice simultaneously [6,9,10]. They used the joint probability of an individual’s health insurance choice and healthcare demand to estimate the distribution of latent variables influencing both decisions and examined the effects of health insurance plans on medical care use by using the MSL estimation method.

However, few studies have examined the relationship between medical care use and bed days at home. To the author’s knowledge, Gilleskie was the first to pay attention to the possibility that rest at home may be a substitute for or a complement to medical care [9]. She tested the substitutability of workers’ absenteeism and medical care use for some acute illness and found that policies restricting medical care use can make them complements for working males.

Few studies have assessed the effects of health insurance on “sick leave” days for individuals outside the labor market as a result of illness. Previous studies have typically focused on lost work days from a worker’s own illness or that of his or her family members [10-13].

In sum, this paper differs from most studies in that it considers both medical care use and bed days at home instead of focusing only on medical care use. Only Gilleskie considered a combination of medical care use and bed days as a choice [9]. This study differs from Gillekie’s in that it focuses on non-working women’s “sick leave” days [9]. The topic of special interest is whether bed days at home are a substitute for or a complement to medical treatment for various medical conditions. This study captures the change in the cost of medical care by differences in the health insurance status that influence the opportunity cost of medical care but not the cost of bed days at home.

## Data and descriptive statistics

We obtained data from the medical condition file of MEPS 2003 [2]. MEPS data are openly available from the U.S Agency of Healthcare Research and Quality(AHRQ) website. The data set included 106,279 conditions for 27,487 individuals. We employed data on medical care use and bed days at home for each illness. For the sample, we selected 12,052 individuals who had only nonpriority medical conditions. In this study, we considered only those bed days at home associated with nonpriority medical conditions under the MEPS definition. That is, we excluded long-term and life-threatening conditions, chronic conditions, and mental health issues. We also excluded those medical conditions related to pregnancy. According to MEPS, long-term and life-threatening conditions include cancer, diabetes, emphysema, high cholesterol, HIV/AIDS, hypertension, ischemic heart disease, and stroke, and chronic conditions include arthritis, asthma, gall bladder disease, stomach ulcers, and all back problems [2,3]. In addition, Alzheimer’s disease or other dementias as well as depression and anxiety disorders are included in the priority list. For a complete list of priority conditions, see MEPS HC-078: 2003 Medical Conditions (2005) [2,3]. We combined the data on individual and family characteristics from the household file of MEPS 2003 with selected data and then identified those observations for non-working married women from the combined data. The ages of individuals in the selected sample ranged from 25 to 64. We restricted the sample to non-working married women for the following two reasons: First, we controlled for the problem of endogeneity between medical care use and health insurance by excluding those married women who were in the labor market because the health insurance status of non-working married women tends to depend on their spouse’s health insurance status or the availability of family coverage. Therefore, we assumed the exogenous provision of health insurance to non-working married women. Second, we determined the behavior of individuals outside the labor market with some illness. If bed days at home among these individuals correspond to sick leave days among the employed, then it is useful to compare these two groups’ behaviors.

This study’s data set was longitudinal because multiple observations for medical conditions were nested within the respondents. However, the number of medical conditions varied across the respondents. That is, we employed an unbalanced panel. The panel dimension of the data was not over time but over the respondent’s medical condition. The use of a longitudinal data set allowed the unobserved heterogeneity of the respondents to be controlled for. Because we assumed that the respondents became ill at random points in time, we considered that they made their decisions on medical care use or bed days at home for each illness on an individual basis. That is, each illness demanded a new decision.

MEPS was useful for this study because the data covered bed days at home and medical care use for each medical condition. Any type of medical use was included in the variable for medical care use, including home health, in-patient, out-patient, office–based, and ER events. For the dependent variable, we considered various combinations of medical care use and bed days for each medical condition for each respondent: no medical care use or bed days (*m* =1), medical care use but no bed days (*m*=2), no medical care use but some bed days (*m*=3), and both medical care use and bed days at home (*m*=4). MEPS records data on various illnesses or nonpriority conditions, only if an individual reports any medical care use or work absences, cut-down days, or bed days was used.

**Figure 1:**
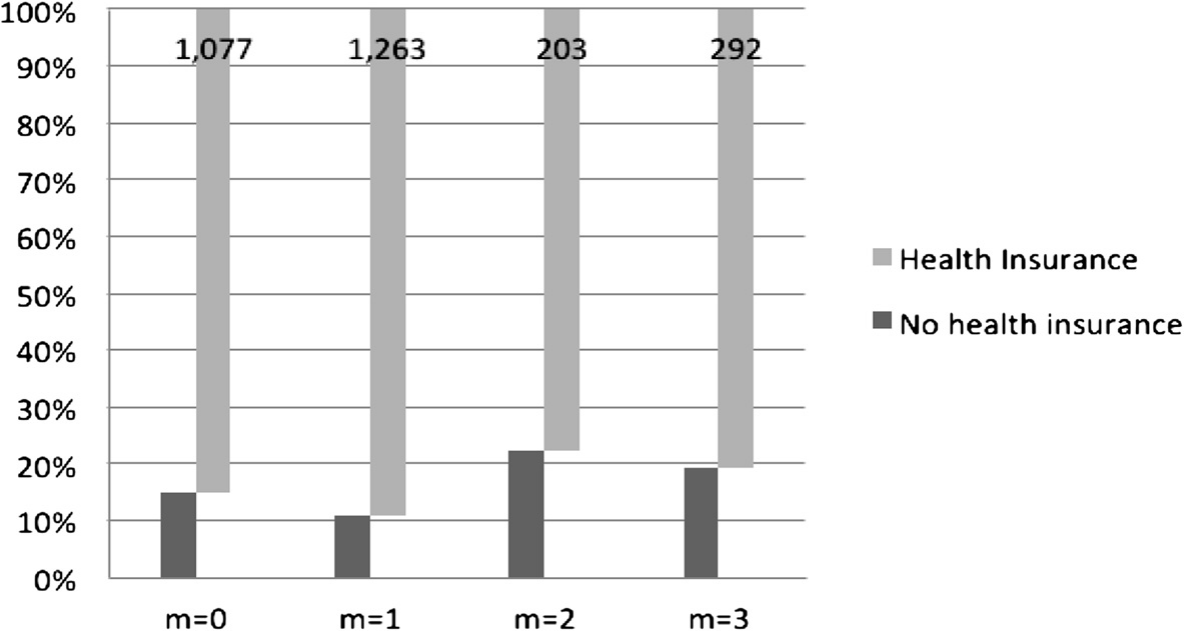
Percentage of nonpriority medical conditions of non-working married women for each combination of medical use and bed days at home.

Table 1 and Figure 1 present the frequency and percentage of nonpriority medical conditions of the respondents, which were classified based on the aforementioned combinations. As shown in Figure 1, those without health insurance accounted for the largest portion of the respondents reporting only bed days at home. The health insurance status played an important role in the respondents’ decision on bed days at home when they had a non-priority illness.

**Table 1:** Frequency of nonpriority medical conditions of non-working married women for each combination of medical care use and bed days at home

**Choice**	**No health insurance**	**Health insurance**	**Total**
No medical care us or bed days (m=0)	187	1,077	1,264
Only medical care use (m=1)	154	1,263	1,417
Only bed-days at home (m=2)	58	203	261
Both medical care use and bed days (m=3)	70	292	362
Total	469	2,835	3,304

Table 2 shows the basic statistics for the dependent variable and covariates. The covariates included the health insurance status, individual factors, and attitudes toward health insurance and medical care use. The health insurance status indicated whether individuals with a medical condition had health insurance. As shown in Table 2, those respondents with nonpriority conditions accounted for approximately 86% of nonpriority conditions. According to attitudes toward health insurance and healthcare services, most medical conditions were related to those respondents who perceived the necessity of health insurance and medical care use.

**Table 2:** Frequency of nonpriority medical conditions of non-working married women for each combination of medical care use and bed days at home

**Variable**	**Mean**	**S. D.**
***Dependent variable***		
Medical care use and bed days at home		
No medical care use or bed days (m=0)	0.38	0.49
Only medical care use (m=1)	0.43	0.49
Only bed days at home (m=2)	0.08	0.27
Both medical care use and bed days (m=3)	0.11	0.31
***Independent variables***		
Health insurance status	0.86	0.35
Private health insurance	0.65	0.48
Public health insurance	0.20	0.40
No health insurance	0.14	0.35
Do not need health insurance		
Disagree strongly	0.83	0.38
Disagree somewhat	0.09	0.28
Uncertain	0.03	0.17
Agree somewhat	0.03	0.17
Agree strongly	0.02	0.14
Can overcome ills without medical help		
Disagree strongly	0.58	0.49
Disagree somewhat	0.20	0.40
Uncertain	0.08	0.27
Agree somewhat	0.13	0.33
Agree strongly	0.02	0.13
Age	48.86	11.16
White (=1 if white)	0.88	0.33
High level of education	0.27	0.45
Moderate level of education	0.49	0.50
Low level of education	0.24	0.42
Family size	3.25	1.54
City/rural area	0.72	0.45
# of children 0-5	0.30	0.65
# of children 6-12	0.37	0.73
# of children 13-18	0.25	0.60
Poverty category		
Poverty 1 (<poverty line)	0.16	0.37
Poverty 2 (100%-125%)	0.06	0.24
Poverty 3 (>125%-200%)	0.15	0.36
Poverty 4 (>200%-400%)	0.29	0.45
Poverty 5 (>400%)	0.34	0.47
Have a chronic condition	0.70	0.46

Note: 3,304 conditions for 925 respondents (aged 24–64).

The variable for priority conditions indicated whether a respondent with a nonpriority condition also had a priority condition. We employed this variable as a proxy for the health status. We included the level of education, age, race, and the place of residence as individual factors. A low level of education indicated those without a high school diploma; a moderate level, those with only a high school diploma; and a high level, those with a bachelor’s degree or more.

We employed the number of children and family size to capture the respondents’ burden of home labor. We divided those children living with the respondents into the following three age groups: 0–5, 6–12, and 13–17 [14].

To capture the economic status, we considered their poverty status, which was classified into the following five groups: negative or poor (less than 100%), near poor (100% to less than 125%), low income (125% to less than 200%), middle income (200% to less than 400%) and high income (greater than or equal to 400%).

## Empirical framework

### Theoretical background

For the framework, we drew on Becker’s theory of time allocation [15,16]. We considered two commodities: health (H) and other commodities (Z_2_). Under this framework, health is produced by the amount of medical care (m) and the amount of time spent on resting (b): H=f_1_ (m, b). The cost of medical care is assumed to have the simple form *a*+*l*_1_*b* , where *a* is a constant and *l*_1_ is the marginal forgone leisure cost per hour spent on bed days at home. Here the cost of medical care is *p*. Non-working married women maximize the utility function *U* = *U*(*H*, *Z*_2_) = *U*(*m*,*b*,*Z*_2_) subject to *a* + *l*_1_*b* + *pm* + *h*(*Z*_2_ )= *S*. The optimal allocation of non-working married women is to consume medical care and bed days at home to meet the condition of equality of the ratio of the marginal products to the ratio of the factor prices: $\frac{\left(U_{H}•f_{m}\right)}{\left(U_{H}•f_{b}\right)}=\frac{p}{l_{1}}$. Here if health insurance reduces the cost of medical care use *p* to *p*_1_ exogenously, then there are two effects that may influence the amount of medical care and the amount of time spent on bed days at home: substitution and income effects.

**Figure 2:**
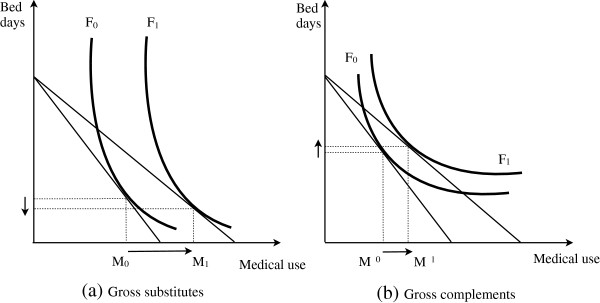
Direction of health insurance effects.

Figure 2 illustrates how the quantity of bed days at home may be influenced by changes in the cost of medical care as a result of health insurance. In both panels, the cost of medical care decreases through the purchase of health insurance because of the shift in the isoquant curve outward from F_0_ to F_1_. In both cases, the amount of medical care increases from M_0_ to M_1_ as a result of a decline in the cost of medical care. However, the two panels show different outcomes for bed days at home. In panel (a), substitution effects are sufficiently large enough such that there is a decrease in the number of bed days at home, implying that medical use and bed days at home can be gross substitutes as a result of changes in the cost of medical care based on the health insurance status. On the other hand, these two goods can be gross complements if hospital visits and bed days at home go together, as shown in Figure 2(b).

### Specification

We assumed the following four medical care options for those respondents with a nonpriority condition: no medical care use bed days; medical care use but no bed days; no medical care use but some bed days; and both medical care use and bed days. We controlled for the unobservable heterogeneity of the respondents by using the MSL estimation strategy [17], see Appendix A] of multinomial logit with unobserved heterogeneity to estimate the effects of health insurance on the respondents’ choice of health care. The multilevel medical condition file for each respondent allowed for the estimation of this unobservable heterogeneity. For the specification, we defined the probability of making a choice conditional on observed characteristics and unobserved individual effects and then derived a simulated likelihood function by drawing pseudo-random unobservable heterogeneity.

Because of the interaction between medical care use and health insurance decisions, we controlled for the selection bias associated with these decisions. We restricted the sample to non-working married women because of the likely dependence of the health insurance status of these women on their spouse’s economic status. In addition, we included attitudes toward health insurance and services in the explanatory variable to control for their endogeneity.

## Results and discussion

### Effects of health insurance

Table 3 shows the multinomial logit results, which indicate that the health insurance status played an important role in the decision on medical care use and bed days at home. Those respondents with health insurance were more likely to use medical care and less likely to consider bed days at home. However, they did not make simultaneous use of medical care and bed days at home. This suggests that if health insurance reduces the cost of medical care relative to bed days at home, then they can be gross substitutes. That is, if medical care use is more expensive than bed days at home for those without health insurance, then they are likely to make sole use of bed days at home when they have a nonpriority condition. This result is consistent with the theoretical implication on the role of relative price in the decision on bed days.

**Table 3:** Multinomial logit results for medical care use and bed days at home

**Choice (based on m=0)**	**Only medical care use (m=1)**			**Only bed days (m=2)**			**Both (m=3)**		
	**Coef.**		**S. E.**	**Coef.**		**S. E.**	**Coef.**		**S. E.**
Health insurance status	0.42	***	0.13	−0.34	*	0.20	−0.20		0.20
Age	−8.4E-04		0.01	−0.03	***	0.01	−0.04	***	0.01
White (=1 if white)	0.48	***	0.14	0.74	***	0.26	0.26		0.21
High level of education	0.08		0.16	0.07		0.27	0.01		0.26
Moderate level education	−0.01		0.13	−0.08		0.23	0.01		0.21
Family size	0.02		0.06	−0.04		0.10	−0.01		0.09
# of children 0-5	−0.07		0.10	−0.24		0.17	−0.64	***	0.18
# of children 6-12	0.01		0.09	0.03		0.15	−0.02		0.14
# of children 13-18	−0.04		0.09	0.10		0.17	−0.05		0.16
City/rural area	0.09		0.11	−0.18		0.19	−0.03		0.19
Have a chronic condition	0.06		0.10	0.03		0.18	0.44	**	0.20
Poverty 2 (100%-125%)	−0.20		0.28	−0.07		0.38	0.19		0.37
Poverty 3 (>125%-200%)	−0.09		0.17	0.34		0.30	0.10		0.29
Poverty 4 (>200%-400%)	−0.04		0.15	0.13		0.23	0.07		0.25
Poverty 5 (>400%)	−0.29	*	0.16	−0.14		0.28	−0.36		0.28
Do not need health insurance									
Disagree somewhat	−0.06		0.30	0.10		0.53	−0.61		0.54
Uncertain	−0.09		0.33	0.17		0.58	−0.58		0.58
Agree somewhat	−0.06		0.40	0.50		0.64	−0.33		0.64
Agree strongly	−0.09		0.37	−0.85		0.70	−0.71		0.64
Can overcome ills without medical help									
Disagree somewhat	−0.08		0.12	−0.47	*	0.25	−0.29		0.21
Uncertain	−0.17		0.19	−0.15		0.31	−0.23		0.26
Agree somewhat	−0.03		0.15	0.08		0.21	−0.43		0.27
Agree strongly	−0.34		0.26	0.28		0.39	−0.64		0.59
Constant	−0.61		0.43	−0.17		0.61	0.47		0.66

* p < 0.10, ** p < 0.05, *** p < 0.01.

Gilleskie investigated medical care use and sick leave days among working males, although these were not the main focus of her study, and found that a policy restricting their access to doctors can reduce their medical care use and absences, suggesting that medical treatment and absences may be complements [9]. However, if the health insurance status of non-working married females corresponds to that of working males such that no health insurance status restricts access to physician visits, then outcomes may have implications different from those suggested in Gilleskie [9].

The results indicate that no health insurance status reduced medical treatment but show an increase in the respondents’ “absences.” In other words, restrictions on medical care use reduced the respondents’ medical treatment but increased their absences. This suggests that medical treatment and absences may be substitutes and that the behaviors of working men and non-working married women may vary according to their healthcare choice. In addition, this implies that health insurance can influence the use of treatment methods other than medical care.

Table 4 shows the results for the multinomial logit model with a random but uncorrelated intercept. The results with the heterogeneity of the respondents controlled for verify that the health insurance status was an important factor influencing the decision on medical care use and bed days at home. The health insurance status had considerable influence on medical care use and bed days at home but not simultaneously, suggesting that they are gross substitutes for non-working married women.

**Table 4:** Results for the multinomial logit model with uncorrelated intercepts for medical care use and bed days at home

**Choice (based on m=0)**	**Only medical care use (m=1)**			**Only bed days (m=2)**			**Both (m=3)**		
	**Coef.**		**S. E.**	**Coef.**		**S. E.**	**Coef.**		**S. E.**
Health insurance status	0.43	***	0.15	−0.37	*	0.22	−0.24		0.23
Age	−2.10E-03		0.01	−0.03	***	0.01	−0.04	***	0.01
White (=1 if white)	0.48	***	0.15	0.70	***	0.27	0.23		0.25
High education	0.13		0.16	0.09		0.26	−0.04		0.28
Middle education	4.20E-04		0.13	−0.02		0.21	0.09		0.22
Family size	0.01		0.06	−0.02		0.09	0.04		0.09
# of children 0-5	−0.08		0.11	−0.25		0.18	−0.73	***	0.20
# of children 6-12	0.02		0.09	0.04		0.14	−0.02		0.15
# of children 13-18	−0.04		0.10	0.10		0.16	−0.08		0.17
City/rural area	0.08		0.11	−0.20		0.18	−0.16		0.19
Have a chronic condition	0.06		0.11	0.01		0.18	0.43	**	0.19
Poverty 2 (100%-125%)	−0.28		0.23	−0.08		0.38	0.22		0.35
Poverty 3 (>125%-200%)	−0.11		0.18	0.35		0.28	0.10		0.30
Poverty 4 (>200%-400%)	−0.05		0.16	0.13		0.27	1.50E-03		0.27
Poverty 5 (>400%)	−0.31	*	0.17	−0.11		0.29	−0.52	*	0.29
Do not need health insurance									
Disagree somewhat	−0.01		0.17	0.03		0.27	−0.02		0.30
Uncertain	0.03		0.28	0.42		0.40	0.24		0.45
Agree somewhat	−0.10		0.27	−1.05	*	0.55	−0.24		0.50
Agree strongly	0.14		0.36	−0.16		0.59	0.33		0.66
Can overcome ills without medical help									
Disagree somewhat	−0.08		0.13	−0.49	**	0.23	−0.12		0.23
Uncertain	−0.18		0.19	−0.18		0.31	−0.12		0.32
Agree somewhat	0.02		0.15	0.12		0.24	−0.34		0.28
Agree strongly	−0.37		0.38	0.38		0.52	−0.56		0.64
Constant	−0.55		0.44	−0.33		0.72	0.09		0.74
Standard Deviation	0.62	***	0.08	−0.79	***	0.17	1.16		0.13

* p < 0.10, ** p < 0.05, *** p < 0.01.

Table 5 shows the results for the multinomial logit model with correlated intercepts. The results based on the easing of the IIA (independence of irrelevant alternatives) assumption verify that health insurance had a positive effect on medical care use and a significant negative effect on bed days at home. The coefficient of health insurance exceeded that in Table 4. The coefficient of health insurance was not significant for the decision on medical care use and bed days at home, suggesting that they are not gross complements for non-working married women.

**Table 5:** Results for the multinomial logit model with correlated intercepts for medical care use and bed days at home

**Choice (based on m=0)**	**Only medical care use (m=1)**			**Only bed days (m=2)**			**Both (m=3)**		
	**Coef.**		**S. E.**	**Coef.**		**S. E.**	**Coef.**		**S. E.**
Health insurance status	0.45	***	0.15	−0.44	*	0.23	−0.30		0.24
Age	−8.10E-04		0.01	−0.03	***	0.01	−0.04	***	0.01
White (=1 if white)	0.48	***	0.15	0.76	***	0.29	0.24		0.27
High education	0.10		0.16	0.21		0.28	0.10		0.29
Middle education	−0.02		0.13	0.12		0.23	0.19		0.23
Family size	0.01		0.06	0.01		0.10	0.07		0.09
# of children 0-5	−0.07		0.11	−0.34	*	0.19	−0.76	***	0.21
# of children 6-12	0.03		0.09	0.04		0.15	−0.02		0.15
# of children 13-18	−0.04		0.10	0.09		0.17	−0.10		0.18
City/rural area	0.06		0.11	−0.15		0.19	−0.10		0.20
Have a chronic condition	0.06		0.11	0.03		0.19	0.43	**	0.20
Poverty 2 (100%-125%)	−0.24		0.23	−0.08		0.39	0.13		0.36
Poverty 3 (>125%-200%)	−0.10		0.18	0.31		0.29	−0.05		0.30
Poverty 4 (>200%-400%)	−0.06		0.16	0.09		0.28	−0.04		0.28
Poverty 5 (>400%)	−0.34	**	0.17	−0.19		0.30	−0.50		0.31
Do not need health insurance									
Disagree somewhat	−0.01		0.17	0.02		0.29	−0.09		0.31
Uncertain	0.02		0.28	0.51		0.42	0.32		0.47
Agree somewhat	−0.13		0.27	−1.06	*	0.57	−0.27		0.50
Agree strongly	0.04		0.36	−0.45		0.61	−0.07		0.60
Can overcome ills without medical help									
Disagree somewhat	−0.06		am	−0.47	*	0.24	−0.15		0.24
Uncertain	−0.14		0.19	−0.17		0.33	−0.15		0.34
Agree somewhat	0.01		0.15	0.15		0.26	−0.29		0.29
Agree strongly	−0.39		0.38	0.54		0.55	−0.38		0.68
Constant	−0.60		0.45	−0.49		0.74	0.09		0.76
Standard Deviation	0.64	***	0.08	1.01	***	0.16	1.28	***	0.12

* p < 0.10, ** p < 0.05, *** p < 0.01.

### Effects of other variables

As shown in Tables 3 and 4, age, race, a priority condition, and living with children under the age of five had significant effects on the decision on medical care use and bed days at home. Younger respondents were less likely to make sole use of bed days at home as well as to make simultaneous use of medical care and bed days at home. White respondents were more likely to make sole use of medical care or bed days at home than receive no treatment.

The results indicate that those with a chronic condition were more likely to make simultaneous use of bed days at home and medical care for a nonpriority condition. This implies that non-working married women with a chronic condition may consider rest and medical care as an effective treatment method for recovering from the illness. Those respondents with children under the age of five were less likely to make simultaneous use of bed days at home and medical care. This suggests that the burden of home labor may limit the simultaneous use of rest and medical care and thus that women with young children may have more difficulty recovering from their illness than other groups. Attitudes toward medical care had a significant effect on the choice of bed days at home. However, attitudes toward health insurance had no significant effect on the choice of medical care and bed days at home.

## Conclusions

Bed days at home among non-working married women are interesting in that they may represent “sick leave” days for individuals outside the labor market as a result of some illness and thus may correspond to lost work days for the employed. Few studies have examined “sick leave” days for individuals outside the labor market as a result of some illness. In this regard, this study contributes to the literature by examining bed days at home and providing important implications for the role of health insurance in the choice of bed days at home as an alternative to medical treatment.

The results suggest that bed days at home and medical care use can become substitutes based on the health insurance status, which is inconsistent with the findings of previous studies suggesting that absences and medical care use among working males are complements when their access to medical care is restricted. This implies that non-working married women are not likely make simultaneous use of rest and medical care to recover from their illness.

Finally, non-working married women with children under the age of five are not likely to make simultaneous use of bed days at home and medical care, which implies that the burden of home labor may restrict their simultaneous use. If their simultaneous use is a better choice for the health status of non-working married women, then the burden of home labor becomes an important health policy issue because it may have considerable influence on their health.

## Appendix A

Controlling for the unobserved heterogeneity of respondents. We define the utility of medical use and bed days at home as

(1) $$U_{\mathrm{imt}}^{*}=\beta_{m}X_{\mathrm{imt}}+\gamma_{1}d_{i1}+\zeta_{\mathrm{im}}+e_{\mathrm{imt}}$$

where *t* is the medical condition; *m* indicates the combination of medical use and bed days at home (m=0: no medical care use or bed days; m=1: medical care use but no bed days; m=2: no medical care use but some bed days; m=3: both medical care use and bed days at home); *X*_*imt*_ denotes individual factors; *d*_*i*_ denotes dummy variable indicating the status of health insurance.

The probability of making choice *m* conditional on observed characteristics *X*_*it*_ that vary across individuals and medical conditions and on unobserved individual effects *ζ*_*i*_ has the following form:

$$P\left(m/X_{\mathrm{it}},\zeta_{i}\right)=\frac{\exp\left(\beta_{m}X_{\mathrm{imt}}+\gamma_{1}d_{i1}+\zeta_{\mathrm{im}}\right)}{1+\sum_{k=2}^{J}\exp\left(\beta_{k}X_{\mathrm{ikt}}+\gamma_{1}d_{i1}+\zeta_{\mathrm{ik}}\right)}$$

The likelihood of making choice m is obtained by integrating out the unobserved variable *ζ*_*i*_, which is assumed to follow a normal distribution, and the integration can be performed numerically instead of analytically:

$$\begin{array}{c} L\left(m_{i}\left|X_{i},\right)\zeta_{i}\right)=\prod_{i=1}^{N}\int \mathrm{Pr}\left(m_{i}\left|X_{i},\zeta_{i}\right)\right)h\left(\zeta_{i}\right)d\zeta_{i}\\ \;\equiv E\left[L\left(m_{i}\left|X_{i},\right)\zeta_{i}\right)\right]\\ \;\approx \frac{1}{S}\prod_{i=1}^{N}\sum_{s=1}^{S}\mathrm{Pr}\left(m_{i}\left|X_{i},\zeta_{\mathrm{is}}\right)\right) \end{array}$$

## Abbreviations

MEPS: Medical expenditure panel survey; MSL: Maximum simulated likelihood; HIE: Rand’s health insurance experiment.

## Competing interests

All authors declare no competing interests.

## Authors’ contributions

CL conceived the study, participated in the design of the study, participated in performing the econometric analysis, and drafted the manuscript. ES participated in the design of the study and helped draft the manuscript. Both authors read and approved the final manuscript.

## Pre-publication history

The pre-publication history for this paper can be accessed here:

http://www.biomedcentral.com/1472-6963/13/243/prepub
